# Reasons Given by ECEC Professionals for (Not) Being in Contact With Parents During the COVID-19 Pandemic

**DOI:** 10.3389/fpsyg.2021.701888

**Published:** 2021-11-08

**Authors:** Fabian Hemmerich, Hande Erdem-Möbius, Lars Burghardt, Yvonne Anders

**Affiliations:** ^1^Chair of Early Childhood Education, University of Bamberg, Bamberg, Germany; ^2^Department of Early Childhood Education, Freie Universität Berlin, Berlin, Germany

**Keywords:** COVID-19, early childhood education and care, ECEC professionals, ECEC managers, professional role understanding, cooperation with parents, social support, pandemic

## Abstract

In response to the COVID-19 pandemic, the German government took drastic measures and ordered the temporary closure of early childhood education and care services (apart from emergency care). Most pedagogical professionals in early childhood education and care (ECEC) settings were unable to provide institutional care for children during this period, and thus experienced difficulties fulfilling their legally mandated professional obligation to educate children. Building on the importance of professional–parent collaboration, this study investigates the reasons ECEC professionals gave for (not) being in contact with parents during the pandemic. The database comprises a nationwide survey conducted between April and May 2020 (*n* = 2,560 ECEC professionals). The results show that the vast majority of respondents were in contact with parents; their stated motives include providing informational or emotional support for parents and children, maintaining a relationship, or inquiring about family wellbeing. The explanations for not being in contact with parents include already existing contact with parents by another member of the ECEC staff, an employer-mandated contact ban, problems on the parents’ side, or personal reasons. We find some differences between managers in center-based childcare, pedagogical employes in center-based childcare, and professionals in family based childcare. Practical implications concerning professional–parent collaboration and the temporary closure of ECEC services are discussed.

## Introduction

By March 2020 at the latest, it was clear that the rapid, worldwide spread of the virus SARS-CoV-2 could no longer be prevented. On March 11, the World Health Organization referred to a pandemic for the first time. The German government, and those of the 16 federal states, continue to take varied and drastic measures to stem the spread of the virus. Among these measures was the temporary closure of center- and family based early childhood education and care (ECEC) services. For at least 2 months from the middle of March 2020 onward, all German ECEC institutions could only offer so-called *emergency care* (*Notbetreuung*), mostly for children whose parents work in systemically relevant professions (e.g., doctors and employes in the food supply sector). The exact duration varied depending on the federal state (Steinmetz et al., 2020).

During this time, most ECEC professionals^1^ were unable to provide institutional care for children and thus experienced difficulties fulfilling their legal mandate to educate the children in their care. Furthermore, they faced challenges in finding new ways to cooperate with parents without face-to-face contact. Existing research emphasizes that strong and supportive relationships with parents positively impact children’s socio-emotional development, educational achievement, and long-term success in school (Jeynes, 2012; Hachfeld et al., 2016; Cohen and Anders, 2020). Accordingly, an examination of the relationship between parents and ECEC professionals during the pandemic^2^ closures is critical to establish insights into its potential effects on families and ECEC professionals.

The present paper aims to understand how and why professionals in center- and family based ECEC services did or did not cooperate with parents during the pandemic. Although some studies have explored the impact of previous pandemics on parents and children (e.g., Bruce-Barrett et al., 2007) and childcare centers (e.g., Shope et al., 2017), little is currently known about the impact of the unprecedented COVID-19 pandemic on ECEC, particularly on the relationships between professionals and parents. Accordingly, this study provides more in-depth insight into why pedagogical professionals were or were not in contact with parents during their services’ temporary closure. In addition to contributing to research on the perspectives and experiences of ECEC professionals when collaborating with parents, the findings can also inform pedagogical practice and policy with the aim of improving the conditions of ECEC settings.

## Overview of the German Early Childhood Education and Care System

The German ECEC system comprises center-based and family based services. Center-based ECEC services are formal institutions where, usually, more than one group of children are cared for and at least two pedagogical employes are responsible for one group. Staff-child ratios vary between 1:3 to 1:5 for groups with 0–3-year-old children and 1:7 to 1:12 for groups with 3–6-year-olds (Autorengruppe Bildungsberichterstattung, 2020). The most important center-based ECEC institutions are preschools (*Kindergärten*) for 3–6-year-old children, crèches (*Krippen*) for 0–3-year-olds, and day care centers (*Kindertagesstätten*) for 0–6-year-olds (Linberg et al., 2013). Family based ECEC services (*Kindertagespflege)*, meanwhile, are official daytime services in the professionals’ or parents’ homes or other suitable premises (Schoyerer et al., 2016). Mostly, a single self-employed professional is responsible for a small group of up to five children. Sometimes, two or more professionals provide joint care for five or more children (Heitkötter and Teske, 2014). In 2019, approximately 92.2% of 3–6-year-old children attended a center-based and 0.7% attended a family based ECEC service. Among children younger than 3 years old, 28.8% attended a center-based and 5.5% attended a family based ECEC service (Autorengruppe Bildungsberichterstattung, 2020).

Approximately 70% of the professionals in center-based ECEC institutions have the official occupation of “educators” (*Erzieherinnen*) and have completed 3–5 years of non-academic vocational training, whereas about 6% of the staff have a bachelor’s degree (Autorengruppe Bildungsberichterstattung, 2020). The managers of center-based ECEC services are also predominantly trained educators (Geiger, 2019). Although the managers have their own autonomy, they are also bound by instructions and financial support from the providers (mostly municipalities or welfare associations). Unlike the staff in center-based ECEC services, the majority of professionals in family based ECEC services have completed only a mandatory basic pedagogical qualification course (Schoyerer et al., 2016). However, legal regulations regarding the level and scope of qualifications vary among the 16 federal states in Germany.

According to §22 SGB VIII (Sozialgesetzbuch – Social Code of Law), both center- and family based ECEC services are legally mandated to support and supplement the education and care of children. Regarding center-based ECEC services, legal educational plans on a state level contain guidelines on how to cooperate with parents; however, these are normally not binding. Some providers have additional guidelines for cooperation with parents. According to §8a of the Social Code of Law, ECEC professionals are also legally obligated to protect children.

## The COVID-19 Pandemic and Professional–Parent Collaboration

The COVID-19 containment measures that limit face-to-face contact have created specific difficulties regarding cooperation between parents and ECEC professionals. Many families have experienced stress due to worries regarding their health, safety, financial situation, and their attempts to balance childcare and work (Andresen et al., 2020; Brown et al., 2020; Cohen et al., 2020; Huebener et al., 2021). Parents of preschool-aged children are even less satisfied with the childcare situation and have encountered additional challenges as their children strongly depend on them (Andresen et al., 2020), resulting in relatively high declines in overall life satisfaction during the pandemic (Huebener et al., 2021). Accordingly, in this period, parents have expressed a desire for support from ECEC institutions for their children’s education (Cohen et al., 2020). The home learning environment has become significantly more challenging because of homeschooling, especially for children from socioeconomically disadvantaged families and families with a migration background (Geis-Thöne, 2020; Ravens-Sieberer et al., 2021). Furthermore, higher parental stress levels during this period can cause a deteriorating family climate, which can negatively affect children’s wellbeing (Geis-Thöne, 2020) and increase the likelihood of child abuse (Brown et al., 2020; Ravens-Sieberer et al., 2021). Oppermann et al. (2021) found that perceived parental stress was the strongest predictor of changes in home learning activities. The more parents felt stressed, the fewer learning activities they undertook with the child. Research has shown that educators are particularly concerned about the increasing stress children have experienced during the pandemic (Jones, 2020). As educators play an essential role in the early detection and reporting of child maltreatment (Fitzpatrick et al., 2020), their ability to assess children’s wellbeing might be limited during the temporary closure of ECEC services. Accordingly, contact with parents is the primary prerequisite for assessing children’s current situations. Scholars have also investigated ECEC professionals’ mental health and emotional stress during the pandemic, finding that pay-cuts, job losses, or preschool closures are the leading economic stressors. In their personal lives, professionals have expressed worries regarding their physical and emotional wellbeing, feelings of loneliness and isolation, and their families (Jones, 2020; Pramling Samuelsson et al., 2020; Tarrant and Nagasawa, 2020). ECEC professionals’ wellbeing can affect their relationships with the children (Whitaker et al., 2015) as well as their cooperation with parents (Kuusimäki et al., 2019).

In general, preschools are ill-prepared for pandemics (Shope et al., 2017; Pramling Samuelsson et al., 2020). Many ECEC professionals lack knowledge and competencies in dealing with pandemics in professional practice, including adopting information and communication technologies (ICT) for educational purposes (Jones, 2020; Pramling Samuelsson et al., 2020; Tarrant and Nagasawa, 2020; Cohen et al., 2021). Langmeyer et al. (2020) found that, even if family households are equipped with ICT, most children in Germany had little contact with their educators. However, Cohen et al. (2021) found that 75% of professionals used ICT for contacting parents, though most of these contacts were sporadic. Institutional rules at the ECEC services, particularly in ECEC centers, have also affected professionals’ relationships with parents, such as data protection or prohibition of usage of personal devices for contacting parents (Studienleitungen “Elementarpädagogik/Elementarbildung” an Österreichs Pädagogischen Hochschulen and Projektteam, 2020). Professionals may also avoid discussing sensitive topics with parents via ICT (e.g., conflicts, health issues) (Kuusimäki et al., 2019) or have privacy and security concerns (Pramling Samuelsson et al., 2020). Parents’ anticipated reactions can also affect their contact. Furthermore, scholars have found that professionals have developed more positive attitudes toward using ICT in pedagogical practice during the pandemic (Cohen et al., 2021). They have adopted creative approaches to contacting parents and children such as providing self-made videos, offering live morning meetings online, or sending emails to parents with ideas for joint activities with their children (Dayal and Tiko, 2020; Tarrant and Nagasawa, 2020). However, COVID-19 has also brought additional challenges in reaching disadvantaged families, such as families with a migration background, due to a lack of personal contact, difficulties in reaching via digital channels, and existing linguistic barriers (Lüken-Klaßen et al., 2020; Studienleitungen “Elementarpädagogik/Elementarbildung” an Österreichs Pädagogischen Hochschulen and Projektteam, 2020). In Germany, intercultural parent–preschool partnership practices considering the support needs of families with a migration background are not widespread (Hachfeld et al., 2016). For instance, exchanging information (in print or digital form) in languages other than German is not common practice (Viernickel et al., 2013). Outreach problems concerning specific groups have been particularly visible in ECEC services during the pandemic (Studienleitungen “Elementarpädagogik/Elementarbildung” an Österreichs Pädagogischen Hochschulen and Projektteam, 2020).

Previous studies have identified a positive impact on the quality of education in preschools resulting from a professional exchange between team members and collaborative team culture (Wertfein et al., 2013; Resa et al., 2018). Although the pandemic has also resulted in confusion and management problems at some ECEC institutions, some managers have applied good practices, such as developing strategic plans to contact families (Fogarty, 2020; Mitchell et al., 2020). The challenges specific to the pandemic might motivate employes of an ECEC institution to collaborate more closely and develop creative and effective forms of cooperation with parents (Studienleitungen “Elementarpädagogik/Elementarbildung” an Österreichs Pädagogischen Hochschulen and Projektteam, 2020). ECEC professionals’ understanding of their professional role may also affect their collaboration with parents (Anders, 2012). Puriola (2002) found that ECEC professionals perceive their work within five frames: educational (e.g., promoting children’s learning), caring (e.g., ensuring children’s wellbeing), managing (e.g., decision-making), practical (e.g., organizing), and personal (e.g., emotions, skills). Conceptions of their own pedagogical role and attitudes toward the responsibility of preschools can affect professionals’ interactions, including collaboration with parents (Anders, 2012). Especially in a difficult period such as the pandemic, ECEC professionals’ understanding of their professional role might significantly affect whether or how they contact parents.

## Theoretical Framework

### The Importance of Cooperation Between Early Childhood Education and Care Professionals and Parents

Collaboration with parents has a long tradition in the German ECEC system; the practice was first propounded by early pedagogues such as Friedrich Fröbel, who created the kindergarten as an institution to supplement the family (Tschöpe-Scheffler, 2018). In Germany, common established forms of cooperation between ECEC professionals and parents include regular talks when parents drop off and pick up their children, individual conversations about the child’s development, or the organization of information evenings on pedagogical topics. Home visits or involving parents in curriculum-related management decisions are less likely (Fröhlich-Gildhoff, 2013; Viernickel et al., 2013; Hachfeld et al., 2016; Cohen and Anders, 2020). Cooperation between professionals and parents has long been considered an indispensable part of pedagogical work with children (Dusolt, 2018), and it represents an important field of action in ECEC (Betz, 2015) and in the conceptualization of ECEC quality (Anders and Roßbach, 2019). Referring to the structural–processual model of pedagogical quality, which is widely used in research on institutional childcare settings, four main dimensions can be identified: structural characteristics (e.g., educator–child ratio), educational beliefs, educational processes (e.g., interactions between children and educators), and networking with families (Kluczniok and Roßbach, 2014). In this model, it is assumed that ECEC services will have a particularly positive effect on children’s development if the institutions do not focus exclusively on the children but also include their families, forming a partnership between professionals and parents (Anders, 2018). Both sides take responsibility for the development of the child and complement or strengthen each other mutually (Textor and Blank, 2014). Dialog and exchange between parents and ECEC professionals thus build a bridge between the family on the one hand and childcare on the other, creating a basis for mutual acceptance and trust (Dusolt, 2018). This has a positive effect on the child. Parents and ECEC professionals can be seen as equal experts for the respective child, considering that they sometimes have different perspectives as they experience the child in different environments (Anders, 2018). Opening up to each other is about making everyday life in center- and family based childcare transparent for families. In addition, parents attribute a high degree of professionalism to pedagogical professionals and seek their advice and guidance. Cooperation between ECEC professionals and parents does not only mean exchanging information about the child’s behavior, development, and upbringing, but also attempting to coordinate educational goals, shape the educational process together, and complement and support each other in the best possible interests of the child. Thus, continuity between practices in childcare and family can be ensured which focuses on the child’s upbringing and development (Cloos, 2018), although there is also a group of parents whose engagement in partnerships with ECEC professionals is considered difficult.

### Professional Competencies of Early Childhood Education and Care Professionals

For qualified pedagogical work, there must be sufficient time for regular cooperation with parents, as well as the ability and willingness of professionals to approach this task. Models of professional competence (e.g., Fröhlich-Gildhoff et al., 2011) emphasize the interplay between disposition and performance. The term disposition refers to the basic principles of action available to a person, whereas performance describes the implementation of individual abilities and skills – i.e., dispositions – in a specific situation and thus refers to actual action (Fröhlich-Gildhoff et al., 2014). Subject-specific and theoretical knowledge play a central role in determining dispositions. To act professionally in a certain situation, professionals must have knowledge relevant to the specific subject at hand, as well as general pedagogical knowledge (Anders, 2012). These theoretical bodies of knowledge are complemented by implicit experiential knowledge; if reflected upon in a professional context, this knowledge can be made explicit. As such, professionals can actively draw on their own experiences, for example when collaborating with parents. This knowledge influences the manner in which professionals perceive and analyze a concrete situation. The willingness to act is significantly influenced by the perception and analysis of the situation, as well as motivation levels. In addition, action-guiding attitudes and values (e.g., individuals’ understanding of their roles as professionals in ECEC) are crucial to determining whether – and in what manner – professionals act in a concrete situation (Fröhlich-Gildhoff et al., 2011).

Transferring these theoretical assumptions to the present study and to the collaboration between ECEC professionals and parents, we characterize the pandemic closures as a specific challenging situation for professionals. The perception of this situation, as well as different circumstances and the own role understanding, can be assigned to the area of disposition on the one hand and – referring to the structural–processual model of quality – to the dimension of educational beliefs on the other hand. Both areas influence the concrete action (processual quality) and manifest in professional–parent collaboration; in this case, the reasons given for (not) being in contact with parents.

### Types of Social Support

Social support can be defined as the process through which social interactions promote health and wellbeing (Cohen et al., 2000). In the context of our study, social support for families can be considered a key component of ECEC professional–parent cooperation. Examining the importance that ECEC professionals placed on providing social support for families during the pandemic-related closure of ECEC services is, therefore, a key concern of this study. The term “social support” is complex and can refer to a variety of actions. There are various approaches for systematizing different types of social support (Cobb, 1976, 1979; House, 1981): The distinction made by House (1981) between *emotional support*, *informational support*, *instrumental support*, and *appraisal support* is one of the most widely used approaches in social support studies (e.g., Hamilton and Sandelowski, 2004; Ostberg and Lennartsson, 2007). Emotional support includes expressions of appreciation, trust, or concern for someone else and empathic behavior in general. Informational support includes making suggestions, giving advice, and sharing knowledge with others. Instrumental support includes actions that directly benefit someone else, such as the provision of goods and services. Appraisal support is characterized by the communication of information that serves another’s self-assessment, e.g., giving constructive feedback. We apply House’s (1981) conceptualization of social support to our data on reasons given for (not) being in contact with parents to examine the importance ECEC professionals place on different forms of social support.

## Research Questions

We pose the following research questions:

RQ1: What reasons do ECEC professionals give for (not) being in contact with parents during the temporary closure of ECEC settings?
RQ2: In what ways do the following three groups – (1) managers in center-based ECEC settings, (2) pedagogical employes in center-based ECEC settings, and (3) professionals in family based ECEC settings – differ in their reasons for (not) being in contact with parents during the temporary closure of ECEC settings?
RQ3: Are there differences in the professionals’ own understanding of their pedagogical role within the stated reasons for (not) being in contact with parents during the temporary closure of ECEC settings?

## Method

### Research Design and Sampling Procedure

This study is based on a nationwide (but not nationally representative) online survey conducted from April 10 to May 24, 2020, with pedagogical professionals in German center- and family based ECEC settings (Cohen et al., 2020). We carried out a convergent parallel mixed method design by collecting both qualitative (open-ended questions) and quantitative (close-ended questions) data within one survey study, and integrating statistical and text analysis (Creswell, 2014). The mixed-method design allowed us to provide comprehensive as well as detailed perspectives of professionals.

Participant recruitment took place via various channels. We emailed the survey to ECEC institutions and providers, requesting that they forward it to their employes, and distributed the link to various online portals and social media groups for ECEC professionals. Applying the snowball principle, we encouraged participants to forward the link to other professionals. Overall, 4,968 professionals participated in the online survey, which included a dichotomous question addressing if they were currently in contact with parents. Depending on their answer, we then asked participants an open-ended question to describe their reasons for being or not being in contact with parents. For our study, we only included professionals who answered one of these two questions. A closer examination of the data revealed that some people who were not actually professionals (e.g., trainees) had filled out the questionnaire. We excluded all such cases. As we focused merely on professionals officially working at the ECEC services when they participated in the survey, we also excluded those on vacation or who stated they were unable to work at that time. This resulted in a final sample size of 2,560 participants (see Supplementary Figure 1 for further details of how the sample size was derived).

Sociodemographic data of the participants are shown in Table 1. The majority of respondents were female (95.2%). On average, the participants were 41.34 years old. Most participants had non-academic vocational training (74.1%), with training as an educator being the most common. Regarding their profession, more than half of the participants were pedagogical employes in center-based ECEC and approximately a quarter were managers of center-based ECEC institutions or professionals in family based ECEC services respectively.

**TABLE 1:** Sociodemographic data of participants.

		Respondents with reasons for having contact with the parents	Respondents with reasons for *not* having contact with the parents	Total
		(*n* = 2,238)	(*n* = 322)	(*n* = 2,560)
Gender	Female	95.5%	93.5%	95.2%
	Male	4.4%	6.5%	4.7%
	Non-binary	0.1%	0%	0.1%

Age	Mean	41.61	39.40	41.34
	SD	11.20	11.29	11.23
	Range	19–67	21–65	19–67

Qualification	*Only* non-academic vocational training	73.6%	77.3%	74.1%
	*Only* academic degree	17.02%	16.1%	16.9%
	Non-academic vocational training *and* academic degree	8.0%	6.2%	7.7%
	No formal qualification	1.4%	0.3%	1.3%

Profession	Managers in center-based ECEC services	25.8%	7.1%	23.5%
	Employes in center-based ECEC services	46.6%	89.1%	52.0%
	Professionals in family based ECEC services	27.5%	3.7%	24.5%

Written informed consent was given by the participants. Participants were informed that they could stop the survey at any time at no disadvantage. The study abided by APA ethical guidelines on conducting studies with human participants. No formal approval from a governing or institutional review board was required for the study (see guidelines provided by the German Research Foundation for the social sciences^3^).

### Data Analysis

#### Qualitative Analysis

We used qualitative text analysis to answer RQ1 by describing professionals’ reasons (not) to be in contact with parents. All participants’ responses to one of the two open questions on reasons for existing or non-existing contact with parents were first saved in a SPSS Statistics 26 dataset. Subsequently, we transferred them into a MAXQDA 2020 dataset to create thematic categories applying qualitative text analysis (Kuckartz, 2014). First, part of the data was independently coded by two researchers (*n* = 100 responses, approximately 3% of the total data) to build main categories. After each researcher had finished the first coding round, they compared and discussed their results and created a new coding scheme. Due to the complexity of responses (many participants named several aspects) and to avoid loss of information, the creation of subcategories (and in some cases sub-subcategories) was afforded special attention in a second round of independent coding. Multiple coding was applied to each response unit, making it possible to assign each response to several categories and subcategories. To cope with this complexity, both coders agreed that when coding each new response, they would first carefully check whether it fit at least one of the categories or subcategories already created. If this was not the case, they created a new (sub-)category for this response. This coding rule can thus be summarized as “as many categories as necessary and as few categories as possible.” In each category, memos were created by providing a brief description of the code and at least one excerpt from the data (see Supplementary Tables 1, 2 for further information on descriptions of main categories and (sub-)subcategories and examples of responses). We ensured that every response was assigned to both the subcategory and its superordinate main category. Conversely, each answer assigned to a main category had to be assigned to at least one of its subordinate subcategories. After finalizing the coding of 100 responses, the assigned codes were compared again; units of coding with no agreement were discussed to reach a consensus between the two coders. Subsequently, we adjusted the coding scheme. To code all further responses we consulted four more coders, explaining our coding scheme to them. Due to the existence of multiple coders, we ensured the documentation of regular updates in the coding scheme. In addition, the entire coding team met regularly to discuss questions, difficulties, or discrepancies, which helped create consensus between coders. During this coding process, we not only created new categories but also removed or combined existing ones. All categories were formed inductively, except six of the main categories (“emotional support of parents/children,” “informational support of parents/children,” and “instrumental support of parents/children”). These categories were formed deductively based on House’s (1981) systematization approach of different social support types as the importance that ECEC professionals attribute to these types was a focal point of our analysis. Subcategories to these deductive main categories were formed inductively. The fourth type of social support mentioned by House (1981), “appraisal support,” could not be identified in our data.

**TABLE 2:** Descriptive data of items used to compute a scale on professionals’ own understanding of their roles regarding cooperation with parents and support of families.

	*N*	Mean	SD	Min	Max	Skewness	Kurtosis
**Items (1 = do not agree at all; 4 = fully agree)**							
(1) I can’t influence what happens in families^1^	2988	2.77	0.77	1	4	0.24	−0.26
(2) Child development (e.g., language development) is currently the sole responsibility of parents^1^	2994	2.87	0.85	1	4	0.34	−0.52
(3) What happens in the families is none of my business^1^	2987	1.74	0.79	1	4	−0.84	0.11
(4) There are more important issues for me right now than collaborating with parents^1^	2911	1.87	0.84	1	4	−0.68	−0.26
(5) Especially during the temporary closure of center-/family based early childhood education and care, I feel obligated to help parents support their children, for example by providing materials	2995	2.98	0.85	1	4	−0.47	−0.45
Scale: professionals’ own understanding of their roles regarding cooperation with parents and support of families (1 = do not agree at all; 4 = fully agree)	2823	2.75	0.55	1	4	−0.32	0.12

*^1^As these four items have a negative wording, they have later been reverse coded to compute the scale on professionals’ own role understanding. All five items are included in the scale “*professionals’ own understanding of their roles regarding cooperation with parents and support of families.*”*

To check the degree of objectivity of our finalized code system, we applied an intercoder agreement measure using MAXQDA. Approximately 10% of the coded responses to each open-ended question—reasons for being in contact with parents (*n* = 211) and reasons for not being in contact with parents (*n* = 29)—were randomly selected using SPSS. These cases were coded by another person who had not been involved in the previous coding procedure. This person was informed about the coding rules that contained definitions and examples for each category. A percentage value for the total agreement between the research team and the second coder regarding the presence or absence of categories was calculated. For each category, matching non-assignments to a response were also counted as matches in this calculation. This resulted in an agreement value of 93.77% for cases where professionals gave reasons for being in contact with parents and an agreement value of 98.33% for cases where professionals gave reasons for *not* being in contact with parents. These values were considered satisfactory and no revision of the category system or re-testing of the intercoder agreement was undertaken.

#### Quantitative Analysis

##### Variables

###### Reasons for (not) being in contact with parents

To answer RQ2 and RQ3, we converted all main categories for reasons for (not) being in contact with parents from our previous qualitative text analysis to dummy variables in SPSS (0 = not present; 1 = present) for statistical examination.

###### Understanding of one’s own professional role

To evaluate how the ECEC professionals understand their own professional role regarding cooperation with parents and social support of families (RQ3), we computed a new scale which shows if the professionals view cooperation with parents and the support of families as part of their role understanding (1 = do not agree at all; 4 = fully agree, Cronbach’s α = 0.69). For 86 cases of the participants who gave reasons for being in contact and for 20 cases of the participants who gave reasons for not being in contact, we could not compute the scale due to missing data; these cases were excluded. This scale comprises the mean of five items which provide information regarding the extent to which professionals consider certain aspects part of their professional role, here related to supporting families and cooperating with parents. The participants were asked to agree or disagree on a 4-point Likert scale for all five items. The items and the descriptions of the scale are presented in Table 2. We later reverse coded the first four variables with negative wording; high scores on the newly computed scale thus correspond to participants who consider cooperation with parents and support of families an integral part of their professional role. We computed a principal component analysis to confirm that the five items load on one factor. The Kaiser–Meyer–Olkin measure of sampling adequacy was 0.75, representing a relatively good factor analysis, and Bartlett’s test of sphericity was significant (*p* < 0.001). An examination of Kaiser’s criteria and the scree-plot yielded the empirical justification for retaining one factor.

##### Procedure

To answer RQ2, we conducted descriptive analyses of the frequencies with which the three different groups of professionals responded to each main category. In addition, we performed chi-square analyses to identify significant differences in the frequency with which these three groups referred to the different categories for reasons to be in contact with parents. However, for the categories of reasons *not* to be in contact with parents, the sample sizes of managers in center-based ECEC services and professionals in family based ECEC services were extremely small. In addition, most chi-square tests for significant differences between the three groups violated the requirement that the expected frequency is less than 5 for no more than 20% of the cells. For this reason, we used Fisher’s exact test instead to test for significant differences between the three groups of professionals among participants who gave reasons *not* to be in contact with parents. In case of a significant group difference, additional *post hoc* tests using the Bonferroni correction were calculated, both for reasons to be in contact and reasons *not* to be in contact with parents.

To answer RQ3, we split the sample into two groups: ones who gave reasons to be in contact with parents and ones who gave reasons *not* to be in contact with parents. The scale “understanding of one’s own professional role” is an ordinal variable and not normally distributed (Shapiro–Wilk test: *p* < 0.001); the reasons they gave are nominal variables. Therefore, we used the Mann–Whitney U (MWU) test to establish whether those who gave a specific reason to be in contact differ from those who did not give the reason regarding the scale “understanding of one’s own professional role.” For better interpretation, we computed the effect size *r* as proposed by Cohen (1988) if the MWU test revealed a significant group difference by dividing the z-value by the square root of the sample size (Fritz et al., 2012). Following Cohen’s guidelines, effect sizes of 0.1 can be interpreted as a small effect, effect sizes of 0.3 as a medium effect, and effect sizes of 0.5 as a large effect (Coolican, 2009). For each stated reason, we computed a separate MWU test.

As the sample sizes for reasons *not* to be in contact with parents are partly in the single-digit range and the smaller sample is partly more scattered than the larger sample, we decided not to compute MWU tests for this sample as the test would lose validity under these conditions. Therefore, regarding the reasons given for *not* being in contact with parents, we kept descriptive comparisons of differences in the “understanding of one’s own professional role” scale between professionals who gave a specific reason and those who did not.

## Findings

The vast majority of participants in our final sample—87.42%—named reasons for being in contact with parents (*n* = 2,238), whereas only 12.58% of participants gave reasons for *not* being in contact with them (*n* = 322). Table 1 reports the descriptive data on sociodemographic characteristics of both subsamples. There were no meaningful differences between participants who gave reasons for being in contact and participants who gave reasons for *not* being in contact with parents regarding gender, age, and qualification. However, regarding their profession, the subsample of professionals who gave reasons *not* to be in contact with parents differed greatly from the other subsample as well as from the total sample, with the vast majority being pedagogical employes in center-based ECEC (89.1%). The mean of 2.75 (SD = 0.55) of the scale on professionals’ own understanding of their role shows that they tend to agree that cooperating with families and supporting families are a part of their own professional role (see Table 2). In the following, we report the results separately for professionals who gave reasons for contact with parents and for professionals who gave reasons for *no* contact with parents. In each section, we first present the main categories that emerged from our qualitative text analysis. We rooted them in comprehensive thematic dimensions, followed by a figure illustrating the main categories and their most relevant associated subcategories (RQ1). Secondly, as these categories were converted into dichotomous variables, we demonstrate statistical analyses by providing the frequencies of the main categories, together with significant differences between (1) managers in center-based ECEC settings; (2) pedagogical employes in center-based ECEC settings; and (3) professionals in family based ECEC (RQ2). Thirdly, we report the differences in professional role understanding regarding cooperation with parents and support of families within the stated reasons for (not) being in contact with parents (RQ3).

### Reasons for Being in Contact With Parents

**FIGURE 1:**
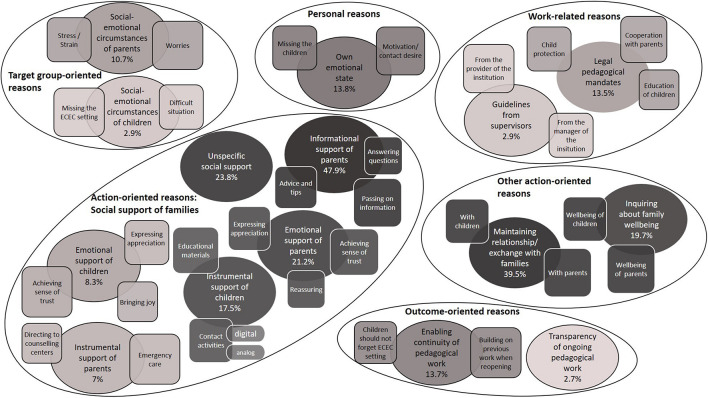
ECEC professionals’ reasons for being in contact with parents. The categories are divided, based on comprehensive thematic dimensions, with large circles. Inside each large circle, the main categories are shown as circles, while subcategories are illustrated as squares. The categories change from dark to light according to the frequency of the assigned responses.

Our final coding system provided 15 main categories (with additional (sub-)subcategories) of the professionals’ reasons for being in contact with parents. These categories were assigned to six thematic dimensions: action-oriented (social support), action-oriented (other), target group-oriented, personal, work-related, and outcome-oriented reasons (see Figure 1). In the following, we present our main categories below each thematic dimension to provide a detailed insight into the professionals’ perspectives.

#### Thematic Dimensions for Being in Contact With Parents and Associated Categories (RQ1)

##### Action-Oriented Reasons: Social Support of Families

The ECEC professionals referred to different types of social support (*informational, emotional, or instrumental*) as their motives for being in contact with parents; nevertheless, many respondents simply mentioned that they wanted to help parents but gave no further details about the nature of this *unspecific social support*. They primarily stated their desire to support parents, but in some cases they also explicitly referred to the children.

*Informational support of parents* was the most mentioned type of social support. Professionals discussed sharing information with parents yet rarely mentioned its content. When they did, it was usually to discuss expectations of when normal care would resume. They also wanted to give advice and tips (e.g., about activities for the children), to answer parents’ questions and be available to them as contact persons. *Emotional support of parents* shows their desire to achieve or maintain a sense of trust among parents. Professionals stressed the importance of making parents aware that they can always rely on them and expressed their appreciation and sympathy to parents. They also wanted to reassure parents when they shared their worries and problems. Furthermore, some underlined the *emotional support of children* by stressing their desire to achieve or maintain a sense of trust among them and express their appreciation to them. In addition, bringing joy to the children (e.g., by sending them Easter gifts) was linked to the intention to distract them from the current difficult situation. *Instrumental support of parents* almost exclusively refers to cases in which professionals mentioned their involvement in providing emergency care. Other aspects, such as directing parents to professional counseling centers were rarely mentioned. *Instrumental support of children* reported by respondents indicates that they provided educational and playing materials for children (e.g., craft templates, coloring books, or literacy or numeracy tasks). A number of professionals also emphasized the importance of being in direct contact with the children, both via “analog” activities (e.g., writing letters) and digital activities (e.g., recording and sending videos or live meetings via video conferencing tools).

##### Other Action-Oriented Reasons

In addition to action-oriented social support motives, we identified two further categories for reasons in which actions on the part of the professionals were in the foreground: *maintaining relationship/exchange with families and inquiring about family wellbeing.* A large proportion of the professionals stated their desire to maintain their relationships and regular exchange with the families. They named specific topics for which a regular exchange was important to them (e.g., the children’s development). In addition, some explicitly reported the necessity of not just a direct relationship with parents but also with children. Moreover, they wanted to detect whether families were doing well during the pandemic by expressing their concerns regarding some parents’ psychological and/or physical stress and the potential adverse effects it has on their children.

##### Target Group-Oriented Reasons

Some professionals gave statements where parents’ or children’s social–emotional circumstances were mentioned, but the professionals’ intention to actively support them or inquire about their wellbeing was not necessarily stated. In these cases, their responses addressed the *social-emotional circumstances of parents*, mentioning parents’ current worries (e.g., regarding their children’s development), extreme parental stress, or strain. Some professionals also mentioned *social-emotional circumstances* of children and underlined how much the children were likely to miss daily life in the ECEC setting or noted that the current situation was fundamentally difficult and stressful for children.

##### Personal Reasons

Some professionals referred to their *own emotional state* to justify their contact with parents. Mostly, their personal motivation or need for contact were mentioned. Some also emphasized how they missed the children.

##### Work-Related Reasons

In two categories, professionals mentioned work-related aspects as reasons for being in contact with parents. Some referred to their *legal pedagogical mandates* and especially stressed cooperation with parents as a central task of their profession. In addition, they noted both their legal mandate of child protection and their legal mandate of educating children. A few professionals referred to *guidelines from their supervisors* (e.g., the provider or the manager of a center-based ECEC service), implying that contact with the parents was more obligatory than voluntary.

##### Outcome-Oriented Reasons

*Enabling continuity of pedagogical work* and *transparency of ongoing pedagogical work* were important outcome-oriented reasons for professionals to be in contact with parents. Professionals emphasized that they wanted to ensure the continuity of their pedagogical work despite the temporary closure of ECEC services. They also hoped that, as soon as all children were able to attend the ECEC facility again, they would be able to build on the extensive collaborations with families that occurred during the temporary closure. Furthermore, many professionals highlighted that children should not forget daily life in the ECEC facility and the professionals who work there. A few professionals stated that through their contact with parents, they wanted to show them that they were still pursuing their professional pedagogical activities instead of just “relaxing.”

#### Frequencies of the Main Categories and Differences Between the Three Groups of Early Childhood Education and Care Professionals (RQ2)

Table 3 depicts the frequencies of the main categories in descending order and the differences between the three groups of ECEC professionals who gave reasons for having contact with parents. *Informational support of parents* was by far the most frequently named reason for existing contact with parents (47.9%), followed by *maintaining the relationship* and *exchange with families* (39.5%). Relatively few professionals (13.5%) justified their contact by the *legal pedagogical mandates*. With less than 3% each, professionals most rarely referred to the *children’s social-emotional circumstances*, *guidelines from supervisors*, and the *transparency of their ongoing pedagogical work* as reasons for their contact. We identified significant differences between the three groups of professionals for 12 of the 15 categories. In six of these cases, the category occurred most frequently, and at the same time significantly more frequently than in both comparison groups, among the managers of center-based ECEC services (e.g., *informational support of parents*, *emotional support of parents, legal pedagogical mandates*). In three cases, the proportion of professionals referring to the category was highest among pedagogical employes in center-based ECEC (*instrumental support of children*, *emotional support of children*, *guidelines from supervisors*). For all three categories, however, *post hoc* tests showed that this proportion differed significantly from professionals in family based ECEC but not from managers in center-based ECEC. In another three cases, the category occurred most frequently among professionals in family based ECEC services. For two of these categories, this proportion was significantly higher than for both comparison groups (*inquiring about family wellbeing*, *enabling continuity of pedagogical work*), and for one category it was only significantly higher than for pedagogical employes in center-based ECEC (*own emotional state*). It is also noteworthy that *legal pedagogical mandates, emotional support of parents* and *unspecific social support* were mentioned significantly less frequently by professionals in family based ECEC services than by both comparison groups.

**TABLE 3:** Main categories and their respective frequencies of reasons for being in contact with parents (*n* = 2,238).

	Frequency					
	
Main category	Managers center-based ECEC	Pedagogical employes center-based ECEC	Professionals family based ECEC	Total	Chi^2^ Test	*Post hoc* Tests (Bonferroni-Correction)
	(*n* = 578)	(*n* = 1,044)	(*n* = 616)	(*n* = 2,238)		
Informational support of parents	60.0%	41.3%	47.9%	47.9%	χ^2^ (2) = 52.412, *p* < 0.001	mc > pc, mc > pf, pf > pc
Maintaining relationship/exchange with families	43.8%	38.0%	38.1%	39.5%	χ^2^ (2) = 5.828, n.s.	/
Unspecific social support	32.5%	24.9%	13.8%	23.8%	χ^2^ (2) = 58.917, *p* < 0.001	mc > pc, mc > pf, pc > pf
Emotional support of parents	27.3%	21.4%	15.3%	21.2%	χ^2^ (2) = 26.030, *p* < 0.001	mc > pc, mc > pf, pc > pf
Inquiring about family well-being	13.3%	20.2%	24.8%	19.7%	χ^2^ (2) = 25.310, *p* < 0.001	pf > mc, pf > pc
Instrumental support of children	17.5%	20.9%	11.7%	17.5%	χ^2^ (2) = 22.707, *p* < 0.001	mc > pf, pc > pf
Own emotional state	13.3%	12.3%	16.9%	13.8%	χ^2^ (2) = 7.110, *p* < 0.05	pf > pc
Enabling continuity of pedagogical work	12.3%	12.1%	17.7%	13.7%	χ^2^ (2) = 11.662, *p* < 0.01	pf > mc, pf > pc
Legal pedagogical mandates	21.1%	13.0%	7.3%	13.5%	χ^2^ (2) = 48.966, *p* < 0.001	mc > pf, mc > pc, pc > pf
Social-emotional circumstances of parents	15.7%	9.8%	7.5%	10.7%	χ^2^ (2) = 23.109, *p* < 0.001	mc > pf, mc > pc
Emotional support of children	8.5%	10.2%	5.0%	8.3%	χ^2^ (2) = 13.360, *p* < 0.01	pc > pf
Instrumental support of parents	11.1%	5.6%	5.5%	7.0%	χ^2^ (2) = 20.223, *p* < 0.001	mc > pf, mc > pc
Social-emotional circumstances of children	2.9%	2.4%	3.7%	2.9%	χ^2^ (2) = 2.467, n.s.	/
Guidelines from supervisors	2.4%	4.5%	0.8%	2.9%	χ^2^ (2) = 19.189, *p* < 0.001	pc > pf
Transparency of ongoing pedagogical work	3.5%	2.7%	1.9%	2.7%	χ^2^ (2) = 2.613, n.s.	/

**n.s.*, *not significant; mc*, *managers center-based ECEC; pc*, *pedagogical employes center-based ECEC; pf*, *professionals family based ECEC*.*

#### Differences in Professionals’ Own Understanding of Their Roles Regarding Cooperation With Parents and Support of Families Within Reasons for Being in Contact With Parents (RQ3)

In the following section, we examine if there are differences in the professionals’ own understanding of their roles regarding cooperation with parents and support of families within their reasons for being in contact with parents. As there were no significant differences between the three groups of ECEC professionals in the variable on professionals’ role understanding, we do not differentiate between these three groups and instead address the total sample of ECEC professionals who gave reasons for having contact with families (*n* = 2,152). Furthermore, we only report differences in their understanding of their professional roles within the main categories which emerged as statistically significant. The scaling of the variable enables the interpretation of mean values larger than 2.5 as (partial) agreement and those smaller than 2.5 as (partial) disagreement. With a mean value of *M* = 2.82 (SD = 0.52), the results show that professionals tend to agree that cooperating with families and supporting families are parts of their understanding of their own role. For the analysis, we computed a MWU Test for each stated reason and compared the role understanding of those who stated that reason with those who did not. The descriptives of those both groups, as well as the results of the MWU test and the calculated effect sizes, are reported in Table 4. Again, we present the reasons in descending order based on their frequency.

**TABLE 4:** Differences in professionals role understanding within reasons for being in contact with parents (*n* = 2,152).

	Professionals’ own understanding of their roles regarding cooperation with parents and support of families (1 = do not agree at all; 4 = fully agree)							
	
Main category	Reason *not* stated			Reason stated			Mann-Whitney-*U*-Test	Effect size
		
	*N*	M (SD)	Median	*N*	M (SD)	Median		
Informational support of parents	1117	2.81 (0.53)	2.80	1035	2.84 (0.50)	2.80	*U* = 565376.00 *Z* = −0.886 n.s.	/
Maintaining relationship/exchange with families	1308	2.76 (0.53)	2.80	844	2.93 (0.48)	3.00	*U* = 450917.00 *Z* = −7.231 *p* < 0.001	*r* = 0.16
Unspecific social support	1634	2.76 (0.53)	2.80	518	3.02 (0.44)	3.00	*U* = 297175.00 *Z* = −10.299 *p* < 0.001	*r* = 0.22
Emotional support of parents	1690	2.79 (0.52)	2.80	462	2.97 (0.47)	3.00	*U* = 310787.00 *Z* = −6.773 *p* < 0.001	*r* = 0.15
Inquiring about family well-being	1731	2.81 (0.52)	2.80	421	2.87 (0.50)	2.80	*U* = 339515.50 *Z* = −2.189 *p* < 0.05	*r* = 0.05
Instrumental support of children	1773	2.81 (0.52)	2.80	379	2.88 (0.51)	3.00	*U* = 312986.00 *Z* = −2.109 *p* < 0.05	*r* = 0.05
Own emotional state	1851	2.81 (0.52)	2.80	301	2.92 (0.51)	3.00	*U* = 246920.00 *Z* = −3.188 *p* < 0.01	*r* = 0.07
Enabling continuity of pedagogical work	1858	2.82 (0.52)	2.80	294	2.87 (0.50)	2.80	*U* = 260248.00 *Z* = −1.310 n. s.	/
Legal pedagogical mandates	1857	2.78 (0.50)	2.80	295	3.07 (0.51)	3.20	*U* = 188323.00 *Z* = −8.693 *p* < 0.001	*r* = 0.18
Social-emotional circumstances of parents	1920	2.80 (0.52)	2.80	232	3.02 (0.49)	3.00	*U* = 167095.50 *Z* = −6.266 *p* < 0.001	*r* = 0.14
Emotional support of children	1973	2.82 (0.52)	2.80	179	2.90 (0.50)	3.00	*U* = 159107.00 *Z* = −2.211 *p* < 0.05	*r* = 0.05
Instrumental support of parents	2001	2.83 (0.52)	2.80	151	2.73 (0.53)	2.80	*U* = 135618.00 *Z* = −2.114 *p* < 0.05	*r* = 0.05
Social-emotional circumstances of children	2090	2.82 (0.52)	2.80	62	2.93 (0.55)	3.00	*U* = 54735.50 *Z* = −2.100 *p* < 0.05	*r* = 0.05
Guidelines from supervisors	2086	2.82 (0.52)	2.80	66	2.78 (0.58)	2.80	*U* = 67478.50 *Z* = −0.275 n. s.	/
Transparency of ongoing pedagogical work	2095	2.82 (0.52)	2.80	57	2.94 (0.46)	3.00	*U* = 52788.00 *Z* = −1.505 n. s.	/

**n.s.*, *not significant*.*

We find statistically significant differences within eleven of the fifteen main categories of reasons for contact with families regarding the professionals’ understanding of their roles. For ten of the reason given by the professionals, the results of the MWU reveal that those who gave the reason find it comparatively more important to cooperate with parents and support families than those who did not give the reason. The reasons are as follows: maintaining relationship/exchange with families (*U* = 450917.00, *Z* = −7.231, *p* < 0.001, *r* = 0.16), unspecific social support (*U* = 297175.00, *Z* = −10.299, *p* < 0.001, *r* = 0.22), emotional support of parents (*U* = 310787.00, *Z* = −6.773, *p* < 0.001, *r* = 0.15), emotional support of children (*U* = 159107.00, *Z* = −2.211, *p* < 0.05, *r* = 0.05), inquiring about families’ wellbeing (*U* = 339515.50, *Z* = −2.189, *p* < 0.05, *r* = 0.05), instrumental support of children (*U* = 312986.00, *Z* = −2.109, *p* < 0.05, *r* = 0.05), professional’s own emotional state (*U* = 246920.00, *Z* = −3.188, *p* < 0.01, *r* = 0.07), fulfilling the legal pedagogical mandates (*U* = 188323.00, *Z* = −8.693, *p* < 0.001, *r* = 0.18), social-emotional circumstances of parents (*U* = 167095.50, *Z* = −6.266, *p* < 0.001, *r* = 0.14), and the social-emotional circumstances of children (*U* = 54735.50, *Z* = −2.100, *p* < 0.05, *r* = 0.05).

We find only one group difference where professionals who gave instrumental support as a reason to be in contact with families find it comparatively less important to cooperate with parents and support families than those who did not give this reason (*U* = 135618.00, *Z* = −2.114, *p* < 0.05, *r* = 0.05).

### Reasons for Not Being in Contact With Parents

**FIGURE 2:**
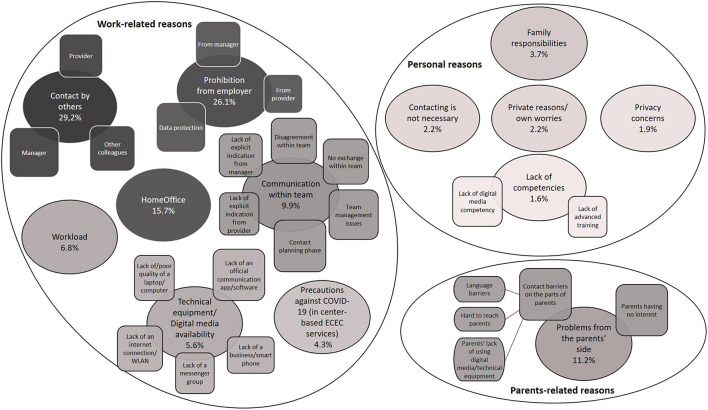
ECEC professionals’ reasons for not being in contact with parents. The categories are divided, based on comprehensive thematic dimensions, with large circles. Inside each large circle, the main categories are shown as circles, while subcategories are illustrated as squares. The categories change from dark to light according to the frequency of the assigned responses.

Our final coding system provided 13 main categories (with additional (sub-)subcategories) for the professionals’ reasons for *not* being in contact with parents. These categories are assigned to three thematic dimensions: work-related, parent-related, and personal reasons (see Figure 2). To delineate professionals’ perspectives and experiences, in the following we present each dimension again with its associated main categories.

#### Thematic Dimensions for *Not* Being in Contact With Parents and Associated Categories (RQ1)

##### Work-Related Reasons

Many professionals noted *contact by others* as a motive for not being in contact with parents themselves. They mentioned that other persons, especially managers, took responsibility for staying in contact with parents. Other pedagogical employes, particularly those who still worked at facilities for emergency care, were also frequently named as responsible contact persons for parents. Fewer respondents reported considering the provider as the responsible actor for being in contact with parents.

Another significant reason was *the prohibition from the employer.* In the context of official prohibitions, the issue of data protection was the most important aspect here. Professionals in center-based ECEC services stressed that they were not allowed to access parents’ contact information outside of their institution. Many professionals named the managers, and some named the providers, as having prohibited contact with parents. Furthermore, the *HomeOffice* situation brought additional challenges to pedagogical practice. Whereas some professionals merely stated working from home in their response, others explained this aspect in further detail. For a number of respondents, having contact with parents was associated merely with face-to-face contact. Some underlined the issue of data protection alongside working from home, mentioning their prohibition from accessing and using parents’ private contact information outside of the ECEC setting.

The unprepared COVID-19 situation in ECEC centers also negatively affected *communication within the team.* The lack of explicit indication from the provider or manager, general management problems within their institutions, and disagreements or lack of exchange within their team were highlighted among respondents. However, some professionals noted being in the preparation phase as a team, working on finding new or better ways to keep in contact with parents.

Some professionals stressed their heavy *workload* during this period. Delivering emergency care was frequently specified to justify not being in contact with parents. In addition, in center-based ECEC services, the *precautions against COVID-19* were seen as a reason not to be in contact with parents, such as not allowing them to enter the facility to avoid face-to-face contact. In addition, *technical equipment* or *digital media availability* were further topics addressed by professionals. They complained that their ECEC institution had failed to provide an official communication app, software, or messenger group that would enable them to contact parents. Furthermore, the poor quality or lack of work-based devices (e.g., laptops, computers, smartphones) or an insufficient internet connection in the ECEC facility were reported.

##### Parent-Related Reasons

Some professionals reported that they wanted contact with parents but were faced with *problems from the parents’ side*. They highlighted difficulties in reaching families, especially ones with a migrant or low socioeconomic background, and mentioned their language barrier, the lack of families’ competencies in using digital media, and the absence of technical equipment in their households. They also mentioned that some parents exhibited no interest in maintaining contact with professionals.

##### Personal Reasons

Professionals also had various personal reasons for not having contact with parents. In addition to their professional life, they also faced challenges in coping with the pandemic in their private life. Among these, *family responsibilities* were remarked upon, such as taking care of their own children or household-related issues. A few professionals reported not being in contact with parents due to *private reasons or worries* such as health concerns for themselves and their families. A small number of professionals found *contacting is not necessary*. Although some opined that families are responsible for the education and care of their children at home, some noted that parents do not require additional support.

We found that a few professionals were also critical about having contact with parents outside of ECEC centers due to *privacy concerns.* They described their hesitance to contact parents via their private telephone numbers or email addresses and underlined the importance of a separation of the professional and private spheres. Furthermore, a *lack of competencies* in contacting parents was reported. Whereas some highlighted their lack of digital media competencies, others mentioned their lack of training in consulting parents in a pandemic situation.

#### Frequencies of the Main Categories and Differences Between the Three Groups of Early Childhood Education and Care Professionals (RQ2)

Table 5 presents the frequencies of the main categories and the differences between the three groups of ECEC professionals who had given reasons for *not* having contact with parents in descending order. Professionals most frequently mentioned that other people were responsible for this. The second most frequent reason given was a prohibition of contacting parents on the part of the employer. Other reasons were given comparatively less frequently. Using the Fisher exact test only showed significant differences in three categories between the three compared groups of professionals. However, additional *post hoc* tests for these three categories only revealed a significant group difference for one category: *Problems from the parents’ side.* Managers in center-based ECEC mentioned this category significantly more frequently than pedagogical employes in center-based ECEC. The inconsistent findings between the Fisher exact tests and the *post hoc* tests using Bonferroni correction may be due to the extremely small case numbers in the two groups of professionals in family based ECEC and managers in center-based ECEC. Overall, the findings on group differences should, therefore, be interpreted with caution.

**TABLE 5:** Main categories and their respective frequencies of reasons for *not* being in contact with parents (*n* = 322).

	Frequency					
	
Main category	Managers center-based ECEC	Pedagogical employes center-based ECEC	Professionals in family based ECEC	Total	Fisher Exact Test	*Post hoc* Tests (Bonferroni-Correction)
	(*n* = 23)	(*n* = 287)	(*n* = 12)	(*n* = 322)		
Contact by others	13.0%	31.0%	16.7%	29.2%	n.s.	/
Prohibition from employer	8.7%	28.6%	0%	26.1%	*p* < 0.01	/
HomeOffice	8.7%	15.7%	16.7%	15.2%	n.s.	/
Problems from the parents’ side	30.4%	8.7%	33.3%	11.2%	*p* < 0.01	mc > pc
Communication within team	8.7%	10.5%	0%	9.9%	n.s.	/
Workload	8.7%	7.0%	0%	6.8%	n.s.	/
Technical equipment/Digital media availability	17.4%	4.5%	8.3%	5.6%	*p* < 0.05	/
Precautions against COVID-19 (center-based ECEC services)	0%	4.9%	0%	4.3%	n.s.	/
Family responsibilities	0%	4.2%	0%	3.7%	n.s.	/
Private reasons/Own worries	4.3%	2.1%	0%	2.2%	n.s.	/
Contacting is not necessary	4.3%	1.7%	8.3%	2.2%	n.s.	/
Private sphere	4.3%	1.4%	8.3%	1.9%	n.s.	/
Lack of competencies	0%	1.7%	0%	1.6%	n.s.	/

**n.s.*, *not significant; mc*, *managers center-based ECEC; ec*, *pedagogical employes center-based ECEC; pf*, *professionals family based ECEC*.*

#### Differences in Professionals’ Own Understanding of Their Roles Regarding Cooperation With Parents and Support of Families Within Reasons for *Not* Being in Contact With Parents (RQ3)

Compared to the professionals who gave reasons for having contact with the families, the professionals who gave reasons not to be in contact identified less with parent cooperation and family support as part of their professional role (*n* = 302, *M* = 2.4, SD = 0.58).

Table 6 presents the descriptives for the ones who gave a specific reason and those who did not give this reason in descending order based on their frequency. Examining the mean values or the medians reveals differences in the professionals’ own understanding of their pedagogical role within some of the stated reasons not to be in contact with parents on a descriptive level. The biggest difference can be found for the reason *contacting is not necessary*; professionals who stated this reason had comparatively lower scores on the scale on cooperation with parents and support of families than professionals who did not state this reason (Mean = 1.83; Median = 1.80 vs. Mean = 2.42; Median = 2.60). In addition, the mean values and medians indicate that professionals who stated that a *prohibition from their employer* was a reason for not being in contact with parents identified more with cooperating with parents and supporting families as part of their professional role than those who did not give this reason (Mean = 2.52; Median = 2.60 vs. Mean = 2.36; Median = 2.40). We found other small differences with respect to the descriptives but these are quite small. Furthermore, the descriptive differences cannot be supported by additional quantitative analyses as the requirements for performing a valid MWU test are not met.

**TABLE 6:** Differences in professionals role understanding within reasons for *not* being in contact with parents (*n* = 302).

	Professionals’ own understanding of their roles regarding cooperation with parents and support of families (1 = do not agree at all; 4 = fully agree)					
	
Main category	Reason *not* stated			Reason stated		
		
	*N*	M (SD)	Median	*N*	M (SD)	Median
Contact by others	215	2.34 (0.60)	2.40	87	2.42 (0.54)	2.60
Prohibition from employer	225	2.36 (0.60)	2.40	77	2.52 (0.52)	2.60
HomeOffice	257	2.40 (0.57)	2.40	45	2.43 (0.65)	2.60
Problems from the parents’ side	266	2.40 (0.58)	2.40	36	2.39 (0.63)	2.60
Communication within team	272	2.40 (0.59)	2.40	30	2.48 (0.54)	2.60
Workload	281	2.42 (0.58)	2.60	21	2.20 (0.58)	2.20
Technical equipment/Digital media availability	284	2.41 (0.59)	2.50	18	2.30 (0.49)	2.40
Precautions against COVID-19 (center-based ECEC services)	288	2.40 (0.58)	2.60	14	2.36 (0.54)	2.30
Family responsibilities	291	2.41 (0.58)	2.40	11	2.22 (0.74)	2.40
Private reasons/Own worries	297	2.40 (0.58)	2.40	5	2.28 (0.84)	2.60
Contacting is not necessary	295	2.42 (0.57)	2.60	7	1.83 (0.82)	1.80
Private sphere	296	2.40 (0.58)	2.40	6	2.30 (0.77)	2.60
Lack of competencies	298	2.40 (0.58)	2.50	4	2.25 (0.52)	2.10

## Discussion

This study, based on data collected through an online survey, aimed to understand how and why professionals in center- and family based ECEC services do or do not cooperate with parents during the pandemic. We applied a mixed-method research design (Creswell, 2014) by analyzing the professionals’ responses to open-ended questions following qualitative text analysis (Kuckartz, 2014) and converting the emergent categories to variables for further quantitative analysis. This allowed us to examine the differences between different groups of ECEC professionals as well as differences in their professional role understanding within the stated reasons.

The findings show that professionals associate reasons for being in contact with parents with various overarching, substantive dimensions. Most of the professionals mentioned action-oriented reasons, particularly providing social support to families. Many aspects mentioned in their responses align with the different types of social support noted by House (1981). To justify contact with parents, professionals referred less frequently to personal feelings, the circumstances of families, and work-related or outcome-oriented aspects.

Informational support of parents and maintaining the relationship and exchange with families emerged as important motives for being in contact. Aspects of informational support were mentioned particularly by managers of center-based ECEC services, indicating that they felt a special responsibility to keep parents informed. Forms of emotional support for parents and instrumental support for parents were also reported comparatively frequently by managers. However, regarding instrumental support, children were addressed more frequently than parents. The instrumental support of parents was mainly referred to in the context of the professionals’ involvement in emergency care provision, which can be mandatory work depending on their employment status. However, instrumental support for children was mainly realized by pedagogical employes in center-based ECEC, and frequently by managers, including different pedagogical activities such as providing craft and learning materials for children or writing letters. In addition, digital media appeared to play a central role as the professionals sent videos and had meetings with the children using video conferencing tools. Mirroring findings from other countries (Dayal and Tiko, 2020; Tarrant and Nagasawa, 2020), our findings illustrate that many professionals are motivated to use ICT for creative and educational contact activities with children. Similarly, Cohen et al. (2021) showed that attitudes toward using ICT have changed positively since the beginning of the pandemic.

Regarding comparisons of the three groups of ECEC professionals, our results indicate that all social support categories are more important motives for existing contact with parents for managers and pedagogical employes in center-based ECEC services than for professionals in family based ECEC services. One possible explanation for this could be that the importance of social support for parents is already emphasized much more strongly in the mandatory staff training in center-based ECEC settings, which is generally more demanding and longer than the usual mandatory qualification course of most professionals in family based ECEC (Schoyerer et al., 2016). Accordingly, in our study we draw attention to the training of professionals, which affects the quality of ECEC services, including cooperation with parents (Anders, 2012).

Another motive for being in contact was inquiring about family wellbeing, which was more frequently stated by professionals in family based ECEC than by managers and pedagogical employes in center-based ECEC. This may be because professionals in family based ECEC are responsible for the care of fewer children and thus probably develop a closer relationship with them and their parents (Viernickel, 2015). Therefore, it is possible that this particular close relationship could make professionals in family based ECEC even more concerned about the families’ wellbeing. Many professionals expressed their concerns that high parental stress levels could endanger the children’s wellbeing. This finding is in line with results from a study in the United States showing similar concerns among early childhood professionals (Jones, 2020). Existing research suggests an increase in domestic violence during the pandemic (Brown et al., 2020; Steinert and Ebert, 2020), so professionals might feel responsible for detecting child maltreatment.

Compared to other aspects, the ECEC professionals in our study referred relatively rarely to official, occupational legal mandates of their pedagogical work to justify contact with parents: cooperation with parents, child protection, or children’s education. Although these legal mandates affect all three groups of ECEC professionals equally, professionals in family based ECEC services referred to them significantly less frequently. As the mandatory qualification course for professionals in family based ECEC is significantly shorter than the usual mandatory training of professionals in center-based ECEC (Schoyerer et al., 2016), the legal foundation of their pedagogical work may play a subordinate role in their training. However, this requires further investigation. Furthermore, as evidenced in cross-country research, professionals’ perspectives may differ based on whether ECEC is a legal right for children and families in the country (Pramling Samuelsson et al., 2020). Therefore, professionals’ perspectives concerning the legal mandate of their pedagogical work deserve more attention by considering different country contexts with different ECEC systems. It must also be emphasized that the comparatively low frequency of this category does not necessarily mean that professionals do not attach a value to the legal mandate of their work. The high rates of the categories *instrumental support of children* and *inquiring about family wellbeing* indicate that many professionals adhere to their legal mandates to educate and protect children even if they do not explicitly mention them.

Furthermore, ECEC professionals’ own understanding of their professional role regarding cooperation with parents and support for families is connected with certain reasons for contact with parents, such as the motive to emotionally support parents and children or to inquire about their wellbeing. Here, the professionals who stated these reasons found it comparatively more important to cooperate with parents and support families than professionals who did not state these reasons. The professional competence model by Fröhlich-Gildhoff et al. (2011) emphasizes that action-guiding attitudes and values—such as professionals’ understanding of their role—influence ECEC professionals’ actions in a concrete situation. Our findings support this assumption as they indicate that a high level of identification with the aspects *cooperation with parents* and *support of families* as components of one’s own professional image is positively correlated to the actual willingness to support parents and children even in times of crisis. We found that among ten of the fifteen reasons for being in contact with parents, those professionals who gave one of the reasons had comparatively higher scores on the scale on role understanding than those who did not state such a reason. It has to be noted, that the means of the scale show that both groups (partially) agree that cooperation with parents and supporting families is part of their role understanding yet the means of the groups who gave reasons are comparatively higher. The effect sizes indicate small effects (Coolican, 2009), indicating that the differences in the role understanding should not be overinterpreted. *Instrumental support of parents* was the only reason where the group comparison revealed lower scores on role understanding for professionals who gave this reason. One possible explanation for this could be that professional role understanding in the context of cooperation with parents and supporting families is negatively connected to organizing and providing emergency care (which is the main aspect of instrumental support of parents). The organization and implementation of emergency care is perceived as obligatory. Accordingly, it can be assumed that those professionals who stated this reason provide (at least partly) emergency care and are therefore in contact with parents, even if they do not find it important to be in contact. This can also be interpreted in the way that professionals who agreed that cooperating with parents and supporting families is an integral part of their role are less likely to give this reason because cooperation beyond emergency care focuses more on voluntary aspects. The positive connections with the professionals’ own understanding of their role and reasons such as maintaining a relationship or inquiring about families’ wellbeing support this interpretation. Among the reasons for not having contact, there are some answers such as *lack of competencies* or *contacting is not necessary* which are in the single-digit percentage range and were mentioned only by a very few professionals. Even though the descriptive group comparison revealed that professionals who said contacting was not necessary (partially) disagreed with seeing cooperation with parents and supporting families as part of their role understanding, this group only consisted of seven participants. This result, as well as other results with small sample sizes, should not be generalized and transferred to the entirety of professionals.

Even though significantly fewer ECEC professionals in our study gave reasons for *not* being in contact with parents during the pandemic, their mentioned aspects provide a vivid picture of their personal and institutional challenges. The most relevant aspects were references to work-related conditions, followed by references to the parents’ circumstances. In contrast, personal reasons were rarely mentioned. The most frequently mentioned reason for refraining from contact with parents was an existing contact by other persons (e.g., the manager of an ECEC facility or other colleagues). This shows that, in some ECEC services, the responsibility for staying in contact with parents lies in the hands of individuals rather than the whole institution. This suggests that some professionals may not have felt responsible for contacting and supporting families during the pandemic closure. This might have been the result of a responsibility diffusion effect among the professionals on an institutional level, meaning that people feel less responsible for their actions when they are part of a group than when alone (Forsyth et al., 2002). Furthermore, the absence of clarity regarding which areas of pedagogical work they feel responsible for (educational, caring, managing, practical, or personal) can affect their responses (Puriola, 2002). Some professionals in center-based ECEC also cited communication problems within their team as a reason for the lack of contact with parents and highlighted insufficient guidelines from their managers, disagreements with their colleagues, and inadequate (or even non-existent) communication within their team. These aspects correspond to other studies which mention challenges related to management and teamwork in ECEC institutions in this period (Fogarty, 2020; Mitchell et al., 2020). Overall, these findings indicate that, at least in some ECEC institutions, there is a need to improve the team culture and management. Corresponding effort and investment would also be worthwhile because professional exchange and a collaborative team culture can positively influence the quality of education in ECEC institutions (Wertfein et al., 2013; Resa et al., 2018).

Another reason given by some professionals to justify the lack of contact with parents was an official prohibition from the employer, which was mentioned mostly by pedagogical employes in center-based ECEC by addressing a prohibition on the part of the manager. Professionals in family based ECEC did not report this aspect, which is not surprising as they are mostly self-employed (Heitkötter and Teske, 2014). As the responses showed, this prohibition in ECEC centers was mostly justified by data protection requirements or guidelines regarding access to parents’ contact information. Data protection and data security are traditionally given high priority in German institutions and, therefore, strongly influence the cooperation between ECEC professionals and families (Cohen et al., 2021). Research has shown that this issue is not specific to Germany; professionals in Austria have described similar challenges (Studienleitungen “Elementarpädagogik/Elementarbildung” an Österreichs Pädagogischen Hochschulen and Projektteam, 2020).

Some respondents cited the poor conditions or the absence of technical equipment such as work computers or smartphones. In addition, they denounced the institutions’ lack of official digital communication tools (e.g., messengers such as WhatsApp). Although professionals would have been willing to contact parents, some hesitated to use their private devices for this purpose. Inadequate technical equipment was frequently mentioned by managers of center-based services, suggesting that the need for action in this regard is often seen primarily by providers. Despite improvements in recent years, many German ECEC centers still lack proper technical equipment (Autorengruppe Bildungsberichterstattung, 2020), which is also reflected in our study. Accordingly, significant steps must be taken to improve the technical conditions of ECEC services.

Approximately one-tenth of the professionals referred to parental aspects to justify non-existing contact. In this context, professionals emphasized that some socially disadvantaged parents were particularly hard to reach, which aligns with other findings (Studienleitungen “Elementarpädagogik/Elementarbildung” an Österreichs Pädagogischen Hochschulen and Projektteam, 2020). Reference was made to the language barriers experienced with parents with non-German mother tongues. Existing pre-COVID-19 findings have already revealed deficits in the manner in which ECEC institutions collaborate with parents with migrant backgrounds (Viernickel et al., 2013; Hachfeld et al., 2016). This problem has been further exacerbated by the loss of face-to-face communication due to the temporary closure of ECEC services. Respondents also highlighted insufficient technical equipment or lack of competencies in using digital media, especially among disadvantaged families. Therefore, it is conceivable that important information (e.g., regarding emergency care) did not reach some parents in time. It is also worth highlighting that, according to numerous respondents, some parents were uninterested in contact. If professionals fail to convince parents that both they and their children would benefit from continued contact in such challenging times, a vicious cycle of mutual disinterest may result. Problems from the parents’ side were mentioned particularly frequently by managers in center-based ECEC.

The personal reasons of professionals are also worth acknowledging, even if they were reported far less than other aspects. Respondents stressed, for example, difficult periods in their private lives resulting from health-related, financial, and socioemotional stress, similar to other studies (Jones, 2020; Tarrant and Nagasawa, 2020). Homeschooling their own children, the difficulties of balancing family and work life, and pay-cuts were some of the reasons given to explain why they were not in contact with parents. Some professionals described feeling incompetent in the use of digital media to collaborate with parents. These findings highlight the importance of recognizing the professionals’ own socioemotional, financial, and professional support needs. A small number of professionals argued that contacting parents is not necessary and that parents are the only actors responsible for their children’s education and care at home, or that the parents are doing well without support from professionals. This finding highlights that it is not just professionals’ own understanding of their pedagogical work (Puriola, 2002) but also in which physical context they feel responsible for pedagogical work that is in question.

Professionals who regarded cooperation and family support aspects as important elements of their professional self-image were less likely to justify a lack of contact with parents by considering it unnecessary and more likely to justify a lack of contact by referring to a prohibition from their employer (but only on a descriptive level). These findings indicate that ECEC professionals’ reluctance to contact parents may not be related to viewing this task as unimportant. The lack of contact was more likely due to work-related circumstances or parental characteristics than to an insufficient sense of responsibility on the part of the professionals.

## Limitations

The following limitations must be addressed. First, it should be emphasized that our study is based on data provided by ECEC professionals and is therefore limited to only one relevant group in the context of cooperation between ECEC professionals and parents. To obtain a more comprehensive picture, reference can be made to a parent survey conducted during nearly the same time period as this study (Cohen et al., 2020; Oppermann et al., 2021). Another limitation regarding our study sample is that there might be a difference between ECEC professionals who worked in emergency care and those who did not, which could not be sufficiently considered here; professionals working in emergency care might have considered their contact with parents more obligatory than voluntary. We decided not to exclude them nevertheless because we assumed that every ECEC professional will have multiple reasons for (not) being in contact with parents and can simultaneously maintain contact obligatorily *and* voluntarily.

Although in our study data were collected to examine the reasons professionals gave for (not) being in contact with parents, no data were collected on the frequency or quality of their contact. This would have been valuable information, especially as professional–parent cooperation should lead to a strong and supportive relationship which indicates high frequency and high-quality interactions. This should be addressed in future studies. Combining reasons for (not) being in contact with parents with their frequency and quality could show what reasons drive the potent cooperation between professionals and parents and therefore also have practical implications as beneficial and hindering reasons could be identified. It should also be noted that no data were collected on cooperation with parents in the period *prior* to the pandemic closure. Furthermore, no data on the importance of cooperation between professionals and parents in an ECEC facility’s mission statement were collected. In addition, the possibility of social desirability in answering the survey questions cannot be excluded. However, as answering these two open questions was voluntary, we do not consider this a serious problem.

Another limitation of this study concerns the coding process. As the researchers’ own interpretations always color qualitative data analysis, we cannot rule out the risk of insufficient objectivity. We attempted to keep this risk as low as possible by creating transparent coding rules for all categories and by regularly discussing and clarifying possible ambiguities. In addition, we calculated a measure of intercoder agreement and obtained very satisfactory values. Consequently, we believe that the objectivity of our code system can be assumed to be sufficiently high.

Regarding the findings on the differences between the three different groups of ECEC professionals in terms of reasons for *not* being in contact with parents, the small sample sizes of managers in center-based ECEC services and professionals in family based ECEC services pose another limitation. Even though the Fisher exact test, which also works for small sample sizes, was calculated in this case, the corresponding findings should be interpreted with caution. This also applies to the differences within the professionals’ reasons for (not) being in contact with parents and their own professional role regarding cooperation with parents and the social support of families. As we described in the method section, the requirements to perform a valid MWU test were not met for the sample of professionals who stated reasons *not* to be in contact with parents. Even though the non-parametric MWU test is suitable for non-normally distributed data, the sample sizes of both groups (professionals who stated a reason and those who did not) differ, especially for reasons not to be in contact. The MWU test loses power if the samples are of different size and loses validity if the smaller sample is more scattered than the larger sample. Because of this, we decided not to carry out this test regarding the professionals’ reasons *not* to be in contact with parent as it may have led to inaccurate results. We therefore decided to stay on a descriptive level with regard to the professionals reasons for *not* being in contact with parents, but even the descriptive results must be interpreted with caution. This especially applies to the small subsamples in the single-digit range as they may represent a very specific (sub-)group of ECEC professionals and should not be generalized.

The small effect sizes of the MWU tests for reasons to be in contact with parents could partly be a result of low variance, with the different “reasons for (not) being in contact with parents” variables being ascribed the binary values of 1 = present or 0 = not present. For future research, it might be interesting to create standardized items based on the categories found in this study. This would avoid the peculiarity of open questions resulting only in those reasons that occur to the professionals at that moment. Thereby, the individual value professionals attach to each of the reasons for (not) being in contact with parents could be assessed more precisely.

Finally, it must be noted that the data collection period of the study was during the first wave of the pandemic in which ECEC services were unprepared for such a challenging situation. Although in summer 2020, relatively normal institutional early childhood education and care was possible due to low infection rates, this situation changed again in fall, as an exponential increase in infection was recorded. As part of renewed measures to contain the spread of the virus, it was decided to temporarily close ECEC facilities again. This closure period lasted from December 2020 to March 2021. As we do not know to what extent professionals’ reasons for (not) being in contact with parents had already changed during this time as a result of the new experiences during the first closure, this would be a starting point for further research.

## Practical Implications

At present, the pandemic is ongoing. Even though it is politically desirable for ECEC services to remain open as long as possible, a further temporary closure of ECEC facilities in Germany cannot be completely ruled out. Consequently, experiences from the first closure period should be used to better prepare for any additional future closures. The findings of our study may be helpful in shaping future effective cooperation between ECEC professionals and parents in such a crisis. Valuable recommendations for action, both on the level of pedagogical professionals and the level of politics, can be derived from our findings.

First, the importance of the joint responsibility of all professionals in an ECEC institution must be emphasized. Especially in large, center-based ECEC services, it is important that each professional is aware of his or her pedagogical responsibility in this regard and does not rely solely on someone else. Managers have a special responsibility in this context. A collaborative team culture characterized by regular exchange based on mutual trust is a key component. If this is not guaranteed from the outset, external team-building measures could be considered. In addition, video conferencing tools can play an important role in ensuring that regular team meetings do not have to be canceled. However, for managers of center-based ECEC services, these circumstances also pose the challenge of ensuring that professionals who only work from home can continue to do pedagogical work (e.g., by creating learning materials or videos for children).

Another practical implication concerns the topic of data protection and data security. To reduce potential insecurities that could hinder action on the part of ECEC professionals, it is important to train them on the legal situation regarding data protection in the context of cooperation with parents. A particular focus should be on informing professionals about what they need to be aware of when using digital media to stay connected with parents. On the policy side, it could be useful to address the extent to which certain data protection laws and policies relevant to the practice of cooperation between professionals and parents can be temporarily relaxed or suspended in times of crisis. In the case of actual changes in data protection laws or policies, the immediate, transparent, and understandable communication of the resulting changes and new opportunities for collaborations to relevant actors in the field of ECEC is important. Because ICT can serve a crucial function in communicating with parents and children during closures, the providers of ECEC services should invest in good technical equipment. Nevertheless, in this context, compliance with data protection requirements must be ensured. Using an ECEC facility’s own software or app as a tool for communicating with parents is generally preferable to using a messenger service such as WhatsApp. In addition, equipping all ECEC professionals with work smartphones can be useful so they do not have to use their private devices. Therefore, the pandemic has confronted us with the necessity of creating new pedagogical concepts, both for collaborating with parents and for providing remote education and care for children.

Finally, the need for innovative and effective approaches for reaching and cooperating with socially disadvantaged parents should be recognized. Professionals should have access to targeted outreach strategies for parents with a migration background and language barriers. Especially at times when face-to-face contact is not possible, it must be ensured that these parents are kept up to date on current events. Distributing information leaflets in the parents’ origin language, employing professional translators, and strengthening inter-agency collaboration between ECEC services and other family support services (e.g., counseling centers and pediatric practices) is important here. It must be underlined, however, that financial support from the providers of ECEC services is necessary to realize such efforts. As the pandemic has made existing social inequalities between families more visible, and can even contribute to their aggravation, efforts to prevent social deprivation should be a high priority for policymakers at the local and national levels.

In conclusion, this paper can broaden the current knowledge of professional–parent cooperation during the COVID-19 pandemic. Furthermore, several constructive suggestions, ranging from the training of ECEC professionals to current pedagogical practice, have been addressed based on professionals’ perspectives. Therefore, collaboration with parents, which is recognized as a component of quality of education in ECEC services (Kluczniok and Roßbach, 2014), should be taken seriously by institutions and policymakers to tackle the negative consequences of the pandemic for educators, families, and children.

## Data Availability Statement

The datasets presented in this article are not readily available because the data are currently reserved for scientific qualifications (Ph.D. and masters’ theses). Requests to access the datasets should be directed to FH, fabian.hemmerich@uni-bamberg.de.

## Ethics Statement

Ethical review and approval was not required for the study on human participants in accordance with the local legislation and institutional requirements. The patients/participants provided their written informed consent to participate in this study.

## Author Contributions

FH and HE-M provided the initial idea for the study, analyzed the qualitative data, coordinated the coding procedure of the qualitative data, and wrote the first draft of the manuscript. FH and LB performed the statistical analyses. LB contributed to the manuscript’s theory section. YA supervised and provided resources. All authors contributed to the conception and design of the study, manuscript revision, and read and approved the submitted version.

## Conflict of Interest

The authors declare that the research was conducted in the absence of any commercial or financial relationships that could be construed as a potential conflict of interest.

## Publisher’s Note

All claims expressed in this article are solely those of the authors and do not necessarily represent those of their affiliated organizations, or those of the publisher, the editors and the reviewers. Any product that may be evaluated in this article, or claim that may be made by its manufacturer, is not guaranteed or endorsed by the publisher.

## Footnotes

1 Unless explicitly stated otherwise, the term “ECEC professionals” always refers to members of the three professional groups: “managers in center-based ECEC services,” “employes in center-based ECEC services,” and “professionals in family based ECEC services.” The term “early childhood teacher” is avoided as professionals in the field of ECEC in Germany are generally not trained as teachers.

2 Unless explicitly stated otherwise, terms such as “pandemic closures” and similar in this article always refer specifically to the period of temporary closures in ECEC services in spring 2020.

3
www.dfg.de/en/research_funding/faq/faq_humanities_social_science/index.html
