# Supplementary material for: Reasons Given by ECEC Professionals for (Not) Being in Contact With Parents During the COVID-19 Pandemic
Source: Front Psychol. 2021 Nov 8;12:701888. doi: 10.3389/fpsyg.2021.701888 (PMC8606416; doi:10.3389/fpsyg.2021.701888)
Supplement: Supplementary file 1 [file Data_Sheet_1.docx]

­­Table 1: Thematic structure of overall dimensions, main categories and subcategories describing reasons of ECEC professionals for being in contact with parents (Note: Some fields in the table are empty because not all main categories have subcategories and not all subcategories have sub-subcategories)

| **Dimension** | **Main Category & Description** | | **Subcategory & Description** | | **Sub-subcategory & Description** | | **Example of Response** |
| --- | --- | --- | --- | --- | --- | --- | --- |
| **Action-oriented reasons: Social support of families** |  | |  | |  | |  |
|  | **Informational support of parents**  Professionals indicate that they want to support families by providing information. | |  | |  | |  |
|  |  | | **Passing on information**  Professionals mention that they want to pass on factual information to parents. | |  | | "To give parents security and information. Be it about care options or activities."  "In addition, passing on current information from the state government and our provider is an important task for us." |
|  |  |  | **Advice and tips**  Professionals mention that they want to give parents actionable advice and tips. | |  |  | "As a professional in family daycare, even before Corona, I was always in regular contact with parents outside of "duty hours". Very often for personal advice around the topic of raising children. This has not changed."  "To give ideas and suggestions for leisure activities" |
|  |  |  | **Answering questions**  Professionals stress that they want to be able to answer questions from the parents’ side. | |  |  | "For questions I am always available for the parents both by phone and by mail."  "I also think it is important to be available for parents who may have questions or may not be able to cope with a situation and are looking for help." |
|  | **Emotional support of parents**  Professionals indicate that they want to support parents on an emotional level. | |  |  |  |  |  |
|  |  |  | **Achieving sense of trust**  Professionals mention that they want to achieve a sense of trust with parents or exude such a feeling towards them. | |  |  | "By listening, understanding and tips you can give the parents the feeling not to be left alone!"  "Maintain contact in order to maintain the educational partnership and trust." |
|  |  |  | **Expressing appreciation**  Professionals mention that they want to express their appreciation to parents and let them know how much they mean to them. | |  |  | "Connection by mail and phone signals to parents and children that they are important to us."  "I also want to show appreciation to parents who are not eligible for emergency care." |
|  |  |  | **Reassuring**  Professionals mention that they want to take care of the parents' worries and problems and reassure them as much as possible by talking to them. | |  |  | "I would like to be informed about the development of the children, to know what the fears and worries of the parents are and possibly encourage them."  "I give parents the chance to talk openly with me and thus discuss frustrations, problems or fears with me and get rid of them." |
|  | **Emotional support of children**  Professionals explicitly indicate that they (also) want to support the parents’ children on an emotional level. | |  |  |  |  |  |
|  |  |  | **Achieving sense of trust**  Professionals explicitly mention that they (also) want to achieve a sense of trust with children or exude such a feeling towards them. | |  |  | "I want to be present for parents and children. I want them to perceive me as a reliable contact person."  "It is important to me to maintain the relationship of trust with the parents and children and not to lose contact with the families." |
|  |  |  | **Expressing appreciation**  Professionals explicitly mention that they (also) want to express their appreciation to children and let them know how much they mean to them. | |  |  | "Communicating to the children that I miss them and that I am thinking of them."  "So that we stay in the minds of the children and they know that we think about them and they are important" |
|  |  |  | **Bringing joy**  Professionals mention that they want to give children pleasure at least for some time. | |  |  | "Also, once a week we drive out something for the children and put mail, tasks, ideas, a self-made game or the Easter nest in front of their door. We want the children to be happy and also have a task and stay in contact with us."  "Providing joy for children and parents is an important part of the educational partnership." |
|  | **Instrumental support of parents**  Professionals indicate that they want to support parents by taking certain actions that directly benefit them. | |  |  |  |  |  |
|  |  |  | **Emergency care**  Professionals mention that they are involved in the provision of emergency care. | |  |  | "I take care of 4 children in emergency care."  "We have a very close relationship and will be providing emergency care starting in May." |
|  |  |  | **Directing to counselling centers**  Professionals mention that they direct parents to (educational) counseling centers or help them make contact. | |  |  | "If additional support is necessary, install further assistance, if necessary, by contacting supportive counseling centers or institutions."  "Through our telephone contact, we can provide relief by listening to the needs of the parents and, if necessary, refer them to counseling services." |
|  | **Instrumental support of children**  Professionals explicitly indicate that they (also) want to support the parents’ children by taking certain actions that directly benefit them. | |  |  |  |  |  |
|  |  |  | **Educational materials**  Professionals mention that they provide families with educational materials for the children | |  |  | "By mail, we provide the children with materials that they can complete at home during this time, such as designing their own portfolio page, coloring pictures, small building projects with parents."  "In addition, we also want to provide children with new learning opportunities to support learning processes." |
|  |  |  | **Contact activities**  Professionals stress being in direct contact with children through certain activities | |  |  |  |
|  |  |  |  |  | **“Analog” contact activities**  Professionals mention direct contact activities with children that do *not* require the use of digital media. | | "We write letters to the children, so they know we are thinking of them."  "Contact with the children via postcards and letters to maintain the relationship and say that you are not alone, and we are happy to support them from afar." |
|  |  |  |  |  | **Digital contact activities**  Professionals mention direct contact activities with children that require the use of digital media | | "I also miss my daycare kids and have already sent them a few videos of me, with small tasks as well."  "Maintaining contact through video calling, so that the little ones can see and hear us, and we are not forgotten and just get a little bit of the children and their current development." |
|  | **Unspecific social support**  Professionals indicate that they want to support or help families, without elaborating on the nature of that support or help. | |  |  |  |  | "It is important to support the families in this special situation as best as we can!"  "Right now, parents need help and support." |
| **Other action-oriented reasons** |  |  |  |  |  |  |  |
|  | **Maintaining relationship/exchange with families**  Professionals indicate that they want to maintain the relationship and/or exchange with families through contact. | |  |  |  |  |  |
|  |  |  | **Maintaining relationship/exchange with parents**  Professionals state that they want to maintain the relationship and/or exchange with the parents through the contact. | |  |  | "Telephone contact to maintain communication."  "The bond with parents and children should be maintained." |
|  |  |  | **Maintaining relationship/exchange with children**  Professionals explicitly state that they (also) want to maintain the relationship and/or exchange with the parents’ children through the contact. | |  |  | "Maintain bond with the children.”  "The exchange with the parents and the children is important to me." |
|  | **Inquiring about family well-being**  Professionals indicate that they want to inquire about the well-being of families during the temporary closure of ECEC services. | |  |  |  |  |  |
|  |  |  | **Well-being of parents**  Professionals indicate that they want to inquire about the well-being of parents. | |  |  | "This means that we have an educational partnership with the parents. Therefore, we regularly contact them, inquire about how they and their children are doing, send information material and tips on what to play, read and learn with the children."  "It is important for us to learn how the parents and children from our facility are doing." |
|  |  |  | **Well-being of children**  Professionals explicitly indicate that they (also) want to inquire about the well-being of the parents’ children. | |  |  | "We want to know how the children are doing, especially in socially disadvantaged or problem families."  "I stay in touch with the families to find out how the kids are doing & what's new." |
| **Target group-oriented reasons** |  |  |  |  |  |  |  |
|  | **Social-emotional circumstances of parents**  Professionals refer to aspects of the social-emotional life circumstances of parents. | |  |  |  |  |  |
|  |  |  | **Worries**  Professionals point out that some parents are currently worried or uncertain. | |  |  | "The parents feel insecure and would like to have information, they would like to be able to exchange information about the children and also about their own situation."  "The parents have many and also worrying questions." |
|  |  |  | **Stress / Strain**  Professionals point out that some parents are currently stressed or overstrained. | |  |  | "From parents who have their children with us in the emergency care, I perceived that they are psychologically overloaded and very happy and have a good feeling, because they know their child in good hands with us."  "Parents are helpless and often overstrained with home office and a child under 3 years old." |
|  | **Social-emotional circumstances of children**  Professionals explicitly (also) refer to aspects of the social-emotional life circumstances of the parents’ children. | |  |  |  |  |  |
|  |  |  | **Missing the ECEC setting**  Professionals indicate that children miss the daily routine at the ECEC institution, the professionals working there, and/or the other children there. | |  |  | "The children miss their preschool and are very happy about mail or whatsapp messages."  "Many children miss the facility." |
|  |  |  | **Difficult situation**  Professionals indicate that the current situation for children is fundamentally difficult. | |  |  | "The children under three years of age do not understand the current situation. I am also an important caregiver who is suddenly no longer available. This puts a strain on the children."  "The break in attachment that has resulted from the current situation is especially bad for the young children involved." |
| **Personal reasons** |  |  |  |  |  |  |  |
|  | **Own emotional state**  Professionals refer to own emotional states. | |  |  |  |  |  |
|  |  |  | **Motivation / contact desire**  Professionals indicate that it is their own need to have contact with families and they are motivated to do so. | |  |  | "Because it is my concern not to leave the families alone and to be there for them."  "Even though I am busy with other issues as a mother myself, it is important to me to make it clear to parents that I am available as a contact person for them and their children." |
|  |  |  | **Missing the children**  Professionals indicate that they miss the children of the ECEC institution. | |  |  | "Children miss me and I miss them too."  "We would like to stay in touch- the children like to come to us and of course we miss them." |
| **Work-related reasons** |  |  |  |  |  |  |  |
|  | **Legal pedagogical mandates**  Professionals refer to work-related legal pedagogical mandates. | |  |  |  |  |  |
|  |  |  | **Cooperation with parents**  Professionals refer to collaboration with parents as one of their legal mandates. | |  |  | "The facility is located in an urban hotspot anyway and collaboration with parents is particularly important there. Only through direct contact and a good educational partnership can families in precarious situations be offered appropriate support."  "We regard parents as those who know their child best and can therefore give us important information about their children, which we take into account in our work. This means that we have an educational partnership with parents." |
|  |  |  | **Education of children**  Professionals refer to education of children as one of their legal mandates. | |  |  | "We want to know how the parents and children are doing in this situation and provide them with material to fulfill at least a minimum of the educational mission."  "In addition, it is important to us to continue to guarantee the educational mission as far as possible." |
|  |  |  | **Child protection**  Professionals refer to child protection as one of their legal mandates. | |  |  | "According to our responsible Youth Welfare Office, only those children may be admitted to emergency care for whom a report of child welfare endangerment has already been made to the Youth Welfare Office. We find this procedure very critical, because we could do a lot for these children BEFORE the child welfare endangerment."  "We also keep an eye on the well-being of the child and are in close contact with socio-pedagogical family support, the youth welfare office and the families concerned." |
|  | **Guidelines from supervisors**  Professionals refer to guidelines from their supervisors regarding the contact with families. | |  |  |  |  |  |
|  |  |  | **Guidelines from the provider of the institution**  Professionals refer to guidelines from the ECEC institutions’ provider. | |  |  | "I am in contact only as much and only in the way that the manager and the provider specify."  "It is very important to the provider that we educators are in contact with the parents, and it is also important to me personally." |
|  |  |  | **Guidelines from the manager of the institution**  Professionals refer to guidelines from the ECEC institutions’ manager. | |  |  | "We were told by the manager to call all the parents of our children and ask how they are doing."  "Requirement of our manager; agreement also with the entire team that at least once a week each child/family is contacted by the respective educator by e-mail." |
| **Outcome-oriented reasons** |  |  |  |  |  |  |  |
|  | **Enabling continuity of pedagogical work**  Professionals indicate that they want to try to ensure continuity of their pedagogical work despite of the temporarily closure of ECEC services. | |  |  |  |  |  |
|  |  |  | **Building on previous work when reopening**  Professionals stress that they want to be able to build on extensive collaborations with families during the temporary closure as soon as all children will attend the ECEC facility again. | |  |  | "For children, a "bridge" for re-entry after closure is to be formed through regular emails with play and activity activities they can do with their parents."  "Maintaining contact is important in order to be able to stay in contact and to make the situation as pleasant as possible for everyone involved after the opening!" |
|  |  |  | **Children should not forget ECEC setting**  Professionals stress that they want to ensure that the children do not forget about the daily life in the ECEC facility during the temporary closure. | |  |  | "Furthermore, it is important to us that we are not forgotten, especially by the children."  "So that the children do not forget us and do not lose touch with the daycare center." |
|  | **Transparency of ongoing pedagogical work**  Professionals stress that they want to achieve transparency for the fact that they are currently working pedagogically. | |  |  |  |  | "But also show the parents that we are now not on paid ‘vacation’ while they have to work double time."  "In the care of under 3-year-olds I find it extremely important that the kids do not forget us and the parents have the feeling that we are always available for them and also that we show interest in the children and do not take a vacation!" |

Table 2: Thematic structure of overall dimensions, main categories and subcategories describing reasons of ECEC professionals for **not** being in contact with parents (Note: Some fields in the table are empty because not all main categories have subcategories and not all subcategories have sub-subcategories)

| Dimension | Main Category & Description | Subcategory & Description | Sub-subcategory & Description | Example of Response |
| --- | --- | --- | --- | --- |
| **Work-related reasons** |  |  |  |  |
|  | **Contact by others**  Professionals indicate that others are responsible for contacting parents. |  |  |  |
|  |  | **Contact by manager**  Professionals indicate that contact with parents is the responsibility of a manager. |  | "The contact is through the manager."  "Is not my responsibility but the order of the manager." |
|  |  | **Contact by other colleagues**  Professionals indicate that other colleagues contact with parents. |  | "This is done by the colleagues who work in emergency care."  "This is what active colleagues do." |
|  |  | **Contact by provider**  Professionals indicate that contact with parents is the responsibility of a provider. |  | "This is uniformly organized by the manager and provider."  "Contact with the parents is taken over by the manager and possibly the provider." |
|  | **Prohibition from employer**  Professionals indicate that their employer does not want them to have contact with parents. |  |  |  |
|  |  | **Prohibition from manager**  Professionals indicate that their manager does not want them to have contact with parents. |  | "It is not wanted by the manager."  "Manager is against contact with parents through social media or the like." |
|  |  | **Prohibition from provider**  Professionals indicate that their provider does not want them to have contact with parents. |  | "It is not encouraged by the provider."  "it is not desired by the provider." |
|  |  | **Data Protection**  Professionals indicate that they cannot directly contact parents due to data protection (e.g., contacting parents from home, using personal devices, accessing contact info from parents). |  | "I do not have email lists (data privacy)"  "The phone numbers of the parents are only in the facility. Email addresses we do not have for data privacy reasons." |
|  | **HomeOffice**  Professionals indicate that they do not have contact with parents because they work from home. |  |  | "I myself work in a home office, but my employees keep in touch with the parents of their groups."  "Since I am almost exclusively home-office and we are not allowed to contact parents from home for privacy reasons." |
|  | **Communication within team**  Professionals indicate that they do not have contact with parents because of communication issues within their team. |  |  |  |
|  |  | **Lack of explicit indication from manager**  Professionals indicate that there is no explicit indication from the manager to contact parents. |  | "There is no mandate from the manager or the provider."  "There are no instructions for action from the manager." |
|  |  | **Lack of explicit indication from provider**  Professionals indicate that there is no explicit indication from the provider to contact parents. |  | "No order received from the provider."  "Up to now it was not planned by the provider." |
|  |  | **Team management issues**  Professionals indicate that it is difficult to get in touch with parents due to management problems in the team. |  | "Since the manager was on vacation and the deputy manager had a lot to organize, it was rather unimportant."  "Our manager quit during this time, so everything is chaotic, the facility has only existed for 6 months." |
|  |  | **Disagreement within team**  Professionals indicate that there is no agreement within the team concerning contacting parents (e.g., contact with parents from other colleagues is not desired). |  | "Some colleagues do not want to contact the parents, so we are not allowed to."  "Large facility, the team is not of the same opinion and the manager does not want you to contact parents." |
|  |  | **No exchange within team**  Professionals indicate that contacting parents has not been discussed in their teams. |  | "There is currently no exchange within the team about how communication could be handled."  "Such things have not been discussed within the team." |
|  |  | **Contact planning phase**  Professionals indicate that they are in the planning phase of contacting parents. |  | "We are currently still working on a concept of how we can help the parents through this difficult time."  "Due to the long duration of the crisis, it is becoming more and more urgent for us to re-establish contact with the parents. We are in the planning phase of how we want to do this." |
|  | **Workload**  Professionals indicate that contact with parents is not possible due to their workload. |  |  | "I am a parent myself and hardly have time to be in contact with parents besides work."  "I don't have space for it. Childcare comes first." |
|  | **Precautions against COVID-19 (in center-based ECEC services**  Professionals indicate that they do not contact parents because of the precautions against COVID-19 in center-based ECEC services (e.g., infection control, curfew). |  |  | "Because of the entry ban, one has too little contact with the parents with whom one could make arrangements or consultations."  "Because of contact ban and a curfew." |
|  | **Technical equipment/Digital media availability**  Professionals indicate that due to the lack of technical equipment and digital media usage in the daycare center, they cannot have contact with parents. |  |  |  |
|  |  | **Lack of an official communication app/software**  Professionals state that they cannot contact parents due to the lack of an app/digital platform in the facility. |  | "Digital possibilities via an app, homepage."  "We were able to contact all parents for the first time on 9.4 through an installed app, it's a pity that we still work like in the stone age." |
|  |  | **Lack of a messenger group**  Professionals state that they cannot have contact with parents due to a lack of using messenger groups. |  | "No WhatsApp group or the like! Only via post."  "Contact via WhatsApp is not common in the institution. Possibly times by phone, if wanted." |
|  |  | **Lack of/poor quality of a laptop/computer**  Professionals indicate that they cannot contact parents due to lack of or poor conditions of a laptop or a computer. |  | "There is only obsolete technology in the daycare center: a PC discarded by the provider and an old laptop. The phone is not a smartphone  "No possibility. Neither PC nor smartphone in the facility." |
|  |  | **Lack of a business/smartphone**  Professionals indicate that they cannot have contact with parents due to a lack of business/smartphones. |  | "I am a part-time and substitute employee and do not have a work cell phone. I do not want to use my personal cell phone."  "No possibility. Neither PC nor smartphone in the facility." |
|  |  | **Lack of an internet connection/WLAN**  Professionals indicate that they cannot contact parents due to a lack of Internet / WLAN in the daycare center. |  | "Technical conditions., e.g. No wifi."  "There is no wifi to use YouTube. Really medieval." |
| **Parent-related reasons** |  |  |  |  |
|  | **Problems from the parents' side**  Professionals indicate that due to problems from the parents' side, they do not contact parents. |  |  |  |
|  |  | **Parents having no interest**  Professionals indicate that parents are not interested in contacting professionals |  | "Lack of interest from parents’ side."  "Facility is located in social hot spot, many parents are not interested." |
|  |  | **Contact barriers on the parts of parents**  Professionals indicate that due to contact barriers on the parts of parents, they do not have contact with parents. |  |  |
|  |  |  | **Language barriers**  Professionals indicate that they cannot contact parents because of parents' language barriers. | "Our parents turn to us rather little. this is due to the high language barrier."  "Unfortunately, I cannot reach e.g. children where the parents do not have German as their first language because of the language barrier." |
|  |  |  | **Hard to reach parents**  Professionals indicate that it is difficult to reach parents (e.g., mail, phone, etc.). | "Mail does not reach all parents."  "Few options, some cannot be reached by phone." |
|  |  |  | **Parents' lack of using digital media/ technical equipment**  Professionals state that they have no contact with parents because parents do not use digital media or have no technical equipment. | "Social hotspot therefore few parents have email."  "We are partially in contact with the parents. With a large part the contact is very difficult, because we have many non-German speaking families. Also digital media are not common with many of our families." |
| **Personal reasons** |  |  |  |  |
|  | **Family responsibilities**  Professionals indicate that due to their family responsibilities, they do not have contact with parents. |  |  | "Am in the home office and take care of my own children."  "Because I work completely in home office to take care of my son, who also does not go to preschool." |
|  | **Private reasons/own worries**  Professionals indicate that they have their concerns and personal problems (e.g., health, financial), so they do not have contact with parents |  |  | "I have to take care of my family and health."  "Since we all only get paid the minimum working hours, I also have little motivation." |
|  | **Contacting is not necessary**  Professionals indicate that contacting parents is not necessary (e.g., parents can take care of their own children). |  |  | "Many parents do not need help. We live in the countryside. The families still get along well."  "I think that parents should be able to take care of their child." |
|  | **Privacy concerns**  Professionals indicate that they do not contact parents because of concerns about their own privacy (e.g., using their private phone number). |  |  | "I also maintain contact with parents exclusively at the daycare center, as I do not mix professional and private matters. I would not even think of contacting parents privately, even if I have known them for a long time."  "I do not want to give my private number to parents." |
|  | **Lack of competencies**  Professionals indicate that because of their lack of competencies, they do not have contact with parents. |  |  |  |
|  |  | **Lack of advanced training**  Professionals indicate that they do not contact parents due to a lack of advanced training. |  | "The basis of the pedagogical work for educators lies in their family-supplementing role. The current situation requires competencies that correspond to the field of systemic family counseling and are therefore difficult for us to provide."  "Lack of further training." |
|  |  | **Lack of digital media competency**  Professionals indicate that they do not have competency in using digital media to contact parents. |  | "Before Corona, I have never worked on the PC. for me, this is all new world."  "Contact is mainly through a chat group on the smartphone. I can not use a smartphone." |

*Figure 1: Flowchart for the formation of the final sample*

Having answered the complete questionnaire and given informed consent (n = 4,968)

Having answered one of the two open questions regarding reasons to be/not to be in contact with parents

(n = 3,213)

Actually being either a manager or pedagogical employee in a center-based ECEC services or a professionals in a family-based ECEC service

(n = 2,653)

Not being on vacation or registered as unable to work currently

(n = 2,560)
